# Controlled interkingdom cell-cell communication between *Saccharomyces cerevisiae* and *Bacillus subtilis* using quorum-sensing peptides

**DOI:** 10.3389/fmicb.2024.1477298

**Published:** 2024-12-12

**Authors:** Tomislav Vološen, Uta Gutbier, Ramón Korn, Juliane Korp, Tobias Göttsche, Linda Schuster, Carolin Pohl, Cindy Rau, Diana Wolf, Kai Ostermann

**Affiliations:** ^1^General Microbiology, Chair of General Microbiology, TUD Dresden University of Technology, Dresden, Germany; ^2^Faculty of Biology, Research Group Biological Sensor-Actuator-Systems, TUD Dresden University of Technology, Dresden, Germany; ^3^Else Kröner Fresenius Center for Digital Health, Faculty of Medicine Carl Gustav Carus, TUD Dresden University of Technology, Dresden, Germany; ^4^Institute of Water Chemistry, TUD Dresden University of Technology, Dresden, Germany

**Keywords:** cell-cell communication, inter-species, signaling peptides, *Bacillus subtilis*, *Saccharomyces cerevisiae*, co-cultivation

## Abstract

Understanding communication among microorganisms through the array of signal molecules and establishing controlled signal transfer between different species is a major goal of the future of biotechnology, and controlled multispecies bioreactor cultivations will open a wide range of applications. In this study, we used two quorum-sensing peptides from *Bacillus subtilis* – namely, the competence and sporulation factor (CSF) and *regulator of the activity of phosphatase RapF* (PhrF)—to establish a controlled interkingdom communication system between prokaryotes and eukaryotes. For this purpose, we engineered *B. subtilis* as a reporter capable of detecting the CSF and PhrF peptides heterologously produced by the yeast *Saccharomyces cerevisiae*. The reporter strain included the ComA-dependent *srfAA* promoter fused to the bioluminescence or fluorescence reporter gene(s) to monitor promoter activity measured in a multimode microplate reader. The first measurements of *srfAA* promoter activity showed a specific response of the reporter strain to the peptides CSF and PhrF. Based on this, systematic mutagenesis of genes that modulate the activity of ComA in the reporter strain resulted in increased activity of the promoter and, thereby, higher sensitivity to the heterologously produced CSF/PhrF. The robustness of the signal transfer was further confirmed in co-cultivation studies in both liquid and solid media. The reporter strain exhibited an up to 5-fold increase in promoter activity in the presence of quorum-sensing peptides-producing cells of *S. cerevisiae*. In summary, a quorum sensing peptide-driven interkingdom crosstalk between yeast and bacteria was successfully established, which might serve as a basis for controlled protein expression in co-cultivations, establishing biological sensor–actuator systems or study cell-cell interaction and metabolite exchange in bioreactors cultivations.

## 1 Introduction

At first glance, the eukaryote baker’s yeast *Saccharomyces cerevisiae* and the prokaryote bacteria *Bacillus subtilis* do not share much with each other. However, both model organisms feature using signal-transducing peptides to influence cellular differentiation processes critical for species survival (López and Kolter, 2010; Michaelis and Barrowman, 2012). For instance, haploid *S. cerevisiae* cells of mating type *α* secrete α-pheromone signaling peptides to the surrounding area, which is crucial for finding cells of the opposite mating type (a-cells) and subsequent fusion of the cells to form a zygote (Michaelis and Herskowitz, 1988). On the contrary, *B. subtilis* developed a network of quorum-sensing peptides vital for the initiation and coordination of cellular differentiation (Magnuson et al., 1994). To establish an interkingdom communication system, we aimed to develop *S. cerevisiae* cells heterologously expressing and secreting active quorum-sensing peptides from *B. subtilis* and to engineer *B. subtilis* cells to generate measurable responses to the quorum-sensing peptides.

For heterologous production and secretion of quorum-sensing peptides from *B. subtilis*, we used *S. cerevisiae* haploid cells that can multiply by budding or can enter the mating (sexual) phase of the life cycle (Michaelis and Barrowman, 2012; Naider and Becker, 2004). In the mating process, two haploid cells of different mating types (*α* or a) recognize each other by expressing and sensing the mating pheromone peptides a or α of the opposite mating type via specific receptors on the cell surface. Activation of the receptors leads to activating the mating kinase cascade and subsequent morphological and genetic changes (Manfredi et al., 1996). The *MFα1* gene encodes four copies of the α-pheromone in a preproprotein necessary for the peptide’s translocation into the endoplasmic reticulum (ER) and subsequent processing of the pheromones. The preproprotein consists of the N-terminal ER-signal peptide and spacer-sequences separating the pheromone single units essential for correct maturation. The indicated ER-signal peptide of *S. cerevisiae* α-mating factor is the most commonly used signal sequence for the secretion of heterologously produced proteins in baker’s yeast (Lin-Cereghino et al., 2013). In this study, we exchanged the sequences of the *MFα1* gene coding for the four copies of the pheromones with sequences coding for quorum-sensing peptides of *B. subtilis.* Although *S. cerevisiae* is not the preferred host for heterologous gene expression in yeast; for example, *Komagataella phaffii* is, we choose this yeast as we have already developed a pheromone-based intra- and interspecies communication system with *S. cerevisiae* (Groß et al., 2011; Hennig et al., 2015).

**Figure 1:**
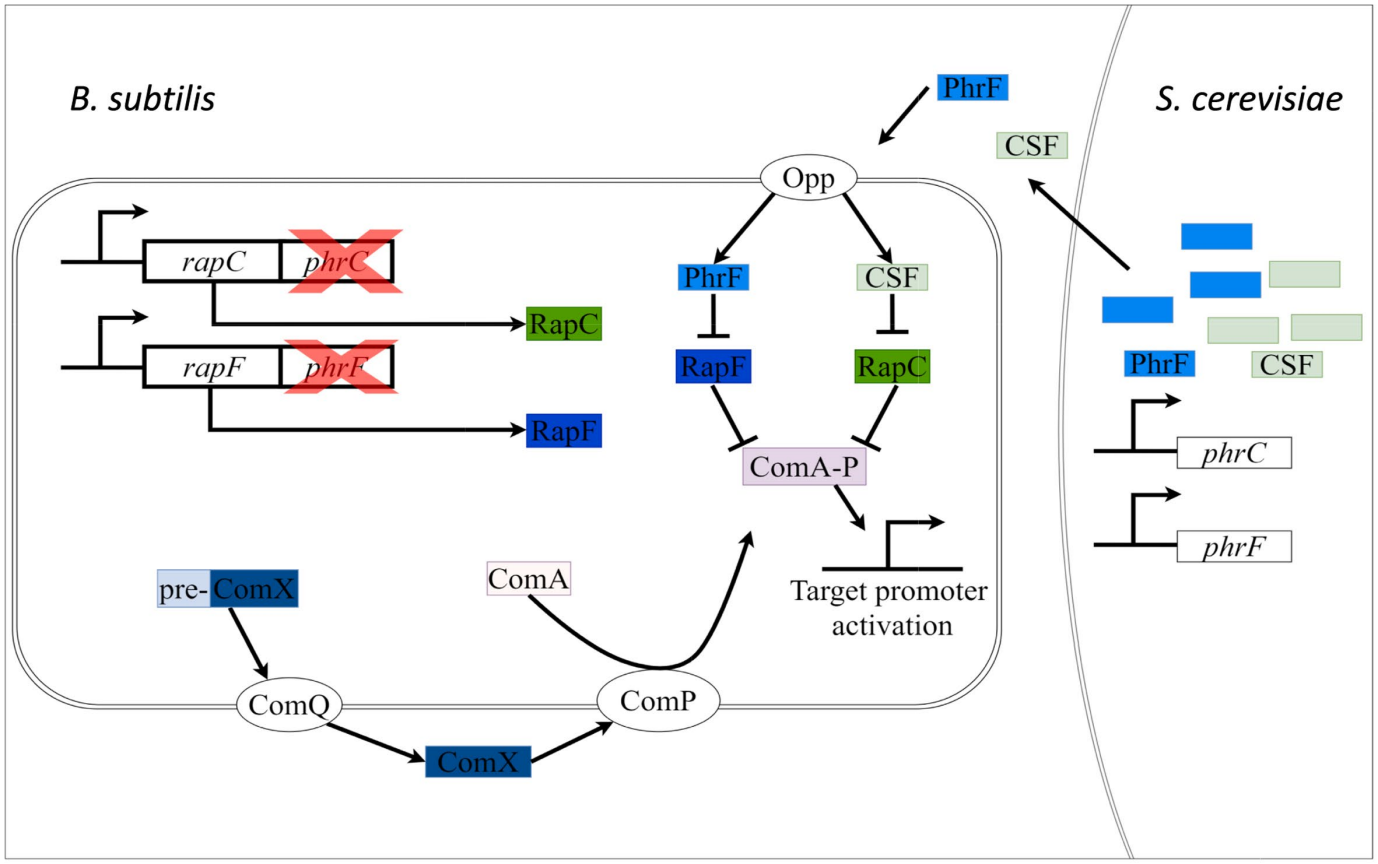
Schematic presentation of cell-cell communication between *S. cerevisiae* and *B. subtilis*. *S. cerevisiae* is engineered to produce and secrete PhrF and competence and sporulation factor (CSF) (PhrC) heterologously. Secreted PhrF and PhrC will be transported via the Opp transporter in the cytoplasmic space of *B. subtilis* ∆*phrC* ∆*phrF.* Heterologously produced peptides will inhibit (perpendiculars; negative effects) RapF and RapC, indirectly activating transcriptional factor ComA. ComA can now activate (arrows; positive effect) the target promoters. We will measure the level of activation by monitoring the promoter activity. Another mode of regulation of ComA is through the ComQXPA system. The peptide ComX is secreted outside the cell with the help of the ComQ membrane protein. ComP, a membrane histidine kinase, is activated by extracellular ComX, which leads to phosphorylation and activation of ComA.

The quorum sensing network in *B. subtilis* is highly developed and provides key environmental, temporal, or positional cues for the initiation and coordination of cellular differentiation (Magnuson et al., 1994). Quorum sensing allows bacteria to communicate, detect, and respond to high cell density (Pottathil et al., 2008). One of the most known processes influenced by quorum-sensing peptides in *B. subtilis* is the development of natural competence (Bongiorni et al., 2005). The transcriptional response regulator ComA mainly regulates natural competence in *B. subtilis*. Once activated, ComA boosts the expression of about 20 genes involved in the competence development of *B. subtilis* (Ogura et al., 2001). Expression of ComA that is part of the *comQXPA* operon is directly modulated by quorum sensing. In the *comQXPA* operon, ComX is an autoinducer quorum-sensing peptide, exported and isoprenylated by the transmembrane transferase ComQ and perceived by the histidine kinase ComP. In the presence of ComX, ComP is activated and transfers phosphate to ComA (Gallegos-Monterrosa and Kovács, 2023). Subsequently, phosphorylated ComA activates its target promoters by interacting with specific binding site sequences in the promoter region (see Figure 1) (Hobbs et al., 2010).

Although the quorum-sensing peptide ComX presents an obvious choice to develop an interkingdom communication system, ComX cannot be heterologously produced in *S. cerevisiae* in an active form due to the essential posttranslational modification by isoprenylation (Okada et al., 2017). Fortunately, *B. subtilis* has another mode of ComA regulation through the Rap-Phr ( **r** esponse regulator **a** spartate **p** hosphatases and **ph** osphatase **r** egulators) family of regulatory phosphatases, which introduces another level of control to major differentiation processes, for example, sporulation, biofilm formation, and natural competence (Gallegos-Monterrosa and Kovács, 2023; Kalamara et al., 2018; Nordgaard et al., 2021). Despite many proteins in the Rap-Phr family, we focus on proteins influencing ComA activity. Rap phosphatases, for example, RapF, RapC, and RapH, inhibit the ComA activity by interacting with the helix-turn-helix DNA-binding domain (Parashar et al., 2013). Indirect activation of ComA is achieved by inhibiting Rap proteins with their cognate Phr peptides PhrF, competence and sporulation factor (CSF) (PhrC), and PhrH (Auchtung et al., 2006). The quorum-sensing peptides PhrF and CSF represent up-and-coming candidates for heterologous expression in *S. cerevisiae* and, therefore, the basis for establishing a cell-cell communication system between yeast and bacteria.

Interkingdom communication in nature is mainly characterized by environmental sensing, quorum sensing, transfer of signaling molecules such as hormones, and host–parasite interaction (Hughes and Sperandio, 2008; Zhou et al., 2017). Probably one of the most complex interkingdom signaling is happening in gut microbiota, where both microbiota and host communicate by the production of the metabolites—both host- and microbiome-derived (Ethridge et al., 2021). More studied interkingdom communication occurs between soil bacteria *Rhizobium* spp. and their symbiotic legume hosts (Calatrava-Morales et al., 2018; Perret et al., 2000). The positive effect of this symbiosis involves the legume production of flavonoids, which activate bacteria production of Nodulation D protein factor and initiation of positive trait nodulation. Nodules contain nitrogen-fixating bacteria for converting atmospheric nitrogen into a form that the plant can use (Hughes and Sperandio, 2008; Perret et al., 2000).

However, application of the synthetic communication by utilizing genetic engineering and synthetic biology is not well-established (Goers et al., 2014). We envision our two-species cultivation synthetic system as an opportunity to study cell-cell interaction and metabolite exchange relevant for co-cultivation in bioprocess-engineered systems such as wastewater treatment plants or the production of chemicals and synthetic proteins. Furthermore, functional interkingdom communication can assist to study and maintain stable fermentation conditions and species co-existence (Jiang et al., 2020). In Figure 1, a schematic overview of the project is presented. We aimed to heterologous express the peptides PhrF and CSF in *S. cerevisiae*. These yeast-produced and -secreted peptides are recognized and transported by the oligo peptide permease (Opp) of the reporter strain *B. subtilis* including deletions of *phrC* and *phrF* (Hughes et al., 2022). Inside the bacterial cell, PhrF and CSF will inhibit their cognate RapF and RapC phosphatases and thereby indirectly activate the transcriptional regulator ComA. The ComA target promoter activity of P*_srfAA_* will then be monitored as a bioluminescence signal in a plate reader, demonstrating the activity and functionality of heterologously produced peptides.

## 2 Materials and methods

### 2.1 Strains and growth conditions

The strains used in this study are listed in Table 1. *B. subtilis* was grown, dependent on its purpose, at 37°C with aeration in Lysogeny broth (Carl Roth, Karlsruhe, Germany) {LB (Luria/Miller [Carl Roth, Karlsruhe, Germany])} (10 g L^−1^ tryptone, 5 g L^−1^ yeast extract, 10 g L^−1^ NaCl) or MNGE media (Carl Roth, Karlsruhe, Germany) {88.2% 1 × MN media (Carl Roth, Karlsruhe, Germany) (1.36% [w/v] dipotassium phosphate × 3 H_2_O, 0.6% [w/v] monopotassium phosphate, 0.1% [w/v] sodium citrate × H_2_O), 1.9% [w/v] glucose, 0.19% [w/v] potassium glutamate, 0.001% [w/v] ammonium ferric citrate, 0.005% [w/v] tryptophan, and 0.035% [w/v] magnesium sulfate}. *S. cerevisiae* was grown at 30°C with aeration, depending on its purpose in yeast peptone dextrose (YPD) media (2% [w/v] peptone, 1% [w/v] yeast extract, 2% [w/v] glucose) or in W0 media (0.17% [w/v] yeast nitrogen base without amino acids, 0.5% [w/v] ammonium sulfate, 2% [w/v] glucose) supplemented with the required amino acids (60 mg L^−1^ L-histidine, 80 mg L^−1^ L-leucine, 20 mg L^−1^ L-methionine, and 30 mg L^−1^ L-lysine). Co-cultivation of *B. subtilis* and *S. cerevisiae* was performed at 31.5°C with aeration in MV media (Carl Roth, Karlsruhe, Germany) (Supplementary Table S1). *Escherichia coli* was grown at 37°C with aeration in LB media. Solid media contained 1.5% (w/v) agar. Selection media for *E. coli* contained ampicillin (100 μg ml^−1^). Selection media for *B. subtilis* contained chloramphenicol (5 μg ml^−1^), erythromycin combined with lincomycin (1 μg ml^−1^ and 25 μg mL^−1^) for MLS or kanamycin (10 μg ml^−1^). Genetic modifications of *E. coli* and *B. subtilis* were performed as described previously (Harwood and Cutting, 1990; Protocols, 2017). *S. cerevisiae* strains were transformed according to Gietz and Woods (2002).

**Table 1:** Strains used in this study.

Strain	Description	Source/reference
*Saccharomyces cerevisiae* BY4741 Δ*bar1*	MATa; *his3*Δ*1 leu2*Δ*0 met15*Δ*0 ura3*Δ*0*; YIL015w::kanMX4	Euroscarf accreditation number Y01408
*S. cerevisiae* NC	BY4741 Δ*bar1* p425GPD	This study
*S. cerevisiae* 4F	BY4741 Δ*bar1* p425GPD-4F	This study
*S. cerevisiae* 4C	BY4741 Δ*bar1* p425GPD-4C	This study
*S. cerevisiae* 2C2F	BY4741 Δ*bar1* p425GPD-2C2F	This study
*Bacillus subtilis* W168	WT, *trpC2*	Laboratory stock
TMB5896	W168 *sacA*::pBS3C*lux*-P*_srfAA_*	This study
TMB5898	W168 *sacA*::pBS3Cl*ux*-P*_srfAA_*, *phrC*::kan	This study
TMB5930	W168 *sacA*::pBS3Cl*ux*-P*_srfAA_*, *phrC*::kan, *phrF*::ery	This study
TMB5931	W168 *sacA*::pBS3Cl*ux*-P*_srfAA_*, *phrC*::kan, *phrH*::ery	This study
TMB6181	W168 *amyE*::pBS1C-P*_srfAA_*-*sf*GFP	This study
TMB6199	W168 *amyE*::pBS1C-P*_srfAA_*-*sf*GFP, *phrC*::kan	This study
TMB6190	W168 *amyE*::pBS1C-P*_srfAA_*-*sf*GFP, *phrF*::ery	This study
TMB6208	W168 *amyE*::pBS1C-P*_srfAA_*-*sf*GFP, *phrC*::kan, *phrF*::ery	This study

### 2.2 Plasmid and strain construction

All primers and plasmids used in this work are listed in Supplementary Tables S2, S3, respectively. General cloning procedures, such as polymerase chain reaction (PCR), restriction enzyme digestion, and ligation, were performed with enzymes and buffers from New England Biolabs^®^ (NEB, Ipswich, MA, United States) according to respective protocols. For DNA amplification, Q5^®^ polymerase (NEB, Ipswich, MA, United States) was used. Positive *E. coli* and *B. subtilis* clones were checked by colony PCR using OneTaq^®^ polymerase. For the construction of the *S. cerevisiae* BY4741 ∆*bar1* strains that produce and secret the peptides CSF and PhrF, the gene sequences of the respective peptides were ordered from BioCat, Heidelberg, Germany. The artificially synthesized sequences were donated in pUC57. The used vector for cloning and genetic modification of *S. cerevisiae*, p425GPD, contains the constitutive *GPD* promoter, resistance cassette for ampicillin (*E. coli*), and the *LEU2* gene as an auxotrophic selection marker for *S. cerevisiae* (Mumberg et al., 1995). All cloning components were digested with *Bam*HI-HF and *Sal*-HF, followed by DNA purification and ligation reaction. The resulting constructs (p425GPD-insert) were verified by sequencing and then transformed with strain *S. cerevisiae* BY4741 ∆*bar1*. To construct *B. subtilis* bioluminescence reporter strains, the used vector pBS3C*lux* harbors the quorum-sensing dependent promoter P*_srfAA_*. Promoter sequences were amplified from *B. subtilis* W168 genomic DNA with primer pairs TM6653/TM6654 and TM6785/TM6786 (Supplementary Table S3). Subsequently, PCR products and backbone vector pBS3C*lux* (Radeck et al., 2013) were digested with *Eco*RI-HF and *Pst*I-HF, followed by DNA purification and ligation. Sequencing confirmed the correctness of inserts in both vectors. Backbone vector pBS1C (Radeck et al., 2013) was used to generate GFP fluorescent reporter strains. The backbone vector was digested with *Eco*RI-HF and *Pst*I-HF, while the fluorescence gene sequence *sf*GFP was cut from the pSB1C3-*sf*GFP vector by using *Xba*I-HF and *Pst*I-HF. Finally, the same promoter P*_srfAA_* PCR product that was used to construct pBS3C*lux* plasmids was digested with *Eco*RI-HF and *Spe*I-HF, allowing promoter fusion to the *sf*GFP and insertion in the pBS1C. Sequencing confirmed successful cloning. Both destination vectors pBS3C*lux* and pBS1C enable the fragment to be integrated into the *sacA* and *amyE* locus of the *B. subtilis* genome (Radeck et al., 2013). To generate strains sensitive to the quorum-sensing peptides CSF and PhrF, we introduced single and double gene deletions in the reporter strains by transforming genomic DNA isolated from deletion strains provided by the Bacillus Genetic Stock Center (BGSC).

### 2.3 Heterologous peptide production in *Saccharomyces cerevisiae*

Expression studies were conducted to analyze the production of the PhrF and CSF in modified strains of *S. cerevisiae* BY4741 ∆*bar1*. Preculture of each strain was prepared in 20 mL of MV media by incubating overnight at 30°C with aeration. The next day, 5 mL of the preculture was inoculated 1:5 in fresh media and incubated for the next 2 h at 30°C with aeration to allow the preculture to homogenize morphologically (day culture). Expression study for each strain started by diluting the day culture to an OD_600_ of 1 in fresh media and incubation at 30°C with aeration again. 2.5 mL samples were taken after culture incubation at 0, 2, 4, 6, and 24 h. OD_600_ of each sample was measured, and the cells were separated from the supernatant by centrifugation at 3,500 *g* for 5 min. 1.6 mL of each supernatant was frozen at −20°C until analyzed by liquid chromatography–mass spectrometry (LC-MS)/mass spectrometry (MS) and Luciferase assay. Expression studies, microscopy, and LC-MS/MS were performed in triplicates.

### 2.4 LC-MS/MS analysis

LC-MS/MS analysis was performed using an ultra-high performance liquid chromatography (UHPLC) system (Nexera, Shimadzu: Shimadzu, Ýuisburg, Germany) consisting of two Nexera X2 LC-30 AD high-pressure pumps (Shimadzu, Ýuisburg, Germany), a Nexera X2 SIL-30 AC autosampler (Shimadzu, Ýuisburg, Germany), a CTO-20 AC column oven (Shimadzu, Ýuisburg, Germany), an ExionLC Degasser (Shimadzu, Ýuisburg, Germany) and CBM-20A controller (Shimadzu, Ýuisburg, Germany). This system was coupled to an electrospray ionization (ESI)–tandem mass spectrometer (triple quadrupole with linear ion trap MS QTRAP 6500+ [Sciex, Madison, United States]) from Sciex. A TSKgel Amide-80 column (TOSOH Biosciences, Griesheim, Germany) (150 × 2.0 mm, 3 μm TOSOH Bioscience [TOSOH Biosciences, Griesheim, Germany]) and corresponding precolumn were used at a constant flow of 0.4 mL min^−1^ at 40°C. The autosampler temperature was set to 15°C. For chromatographic separation, a linear gradient of eluent A (5% [v/v] Acetonitrile, 95% water with 0.125% [v/v] formic acid and 10 mM ammonium formate) and eluent B (95% [v/v] ACN, 5% water with 0.125% [v/v] formic acid) was generated. After an initial stage of 7 min at 80% eluent B, the amount of eluent B was decreased linearly to 60% within 30 s and kept constant at that level for 2 min. Within the next 30 s, the content of eluent B was further decreased to 10% and kept constant for 2 min before returning to the starting conditions within 10 s. The re-established initial conditions were kept constant for another 4.9 min to equilibrate the column. The injection volume was set to 10 μL. Detection of peptides was performed in ESI(+) mode according to optimized ionization conditions for each analyte using the following source conditions: Ion spray +5,500 V, Temperature 400°C, curtain gas 40 psi, collision gas medium, gas 1 and gas 2 at 50 psi. Time dependent changes were monitored by multiple reaction monitoring (MRM) measurements (Supplementary Table S5). Data were analyzed using Analyst software version 1.7 of MultiQuantTM (Sciex, Madison, United States). For quantification, calibration was performed with a synthetic peptide mixture in the concentration range of 0.01–1.00 μM. Each calibration point contains a 150 μL peptide mixture and 10 μL internal standard, including the isotopically labeled peptides of CSF and PhrF in a concentration of 10 μM and *α*-factor at 5 μM, in 50% (v/v) ACN, and 50% H_2_0 + 0.125% (v/v) formic acid. For peptide analysis, 75 μL of the supernatant was mixed with an equal volume of acetonitrile, and 10 μL of internal standard was added afterward. The isotopically labeled peptides, CSF (ER(13C6;15 N4)GMT) (Peptide Specialty GmbH, Heidelberg, Germany) and PhrF (QR(13C6,15 N4)GMI) (Peptide Specialty GmbH, Heidelberg, Germany) were purchased by Peptide Specialty Laboratories (Peptide Specialty Laboratories GmbH, Heidelberg, Germany) GmbH (> 95%). Internal standards and peptide solutions were stored at −18°C. The mixed solutions containing ACN were stored at 6°C.

### 2.5 Luciferase assay

The protocol for measuring the luciferase activity in *B. subtilis* strains carrying *luxABCDE* operon was adopted from the studies by Popp et al. and Radeck et al. The bioluminescence assay was performed in a Synergy Neo3 Hybrid Multimode Microplate Reader from BioTek (Winooski, VT, United States). The plate reader was controlled by the software Gen5™ (Bio Tek, Agilent, Winooski, VT, United States). The procedure in brief: An overnight culture was prepared in the LB media with the required antibiotics. Day cultures, without antibiotics, were fixed to an OD_600_ of 0.05 in the MNGE/MV media and grown until an OD_600_ of 0.15–0.25. Subsequently, the cells were diluted to an OD_600_ of 0.05 and 100 μL of cells per sample were transferred into a 96-well plate (black walls, clear bottom, Greiner Bio-One, Frickenhausen, Germany). Plate reader measurements were done by recording OD_600_ and the luminescence every 5 min over at least 18 h during incubation of the plate at 37°C with agitation. The induction of the cells with synthetic peptides CSF and PhrF and the yeast supernatant was performed at the start or after one or 4 h of cultivation. Luciferase activity (relative luminescence units [RLU]/OD_600_) was defined as the RLU normalized to OD_600_ corrected by medium blank at each time point.

### 2.6 Co-cultivation of *Bacillus subtilis* and *Saccharomyces cerevisiae*

Co-cultivation was performed to show the biological activity of the heterologously produced quorum-sensing peptides on the fluorescence *B. subtilis* P*_srfAA_*-*sf*GFP ∆*phrC* ∆*phrF* reporter strain. Due to the nonlinear correlation between optical density and the number of cells between *B. subtilis* and *S. cerevisiae*, single cultures were analyzed to develop a calibration curve between optical cell density and the number of cells. The calibration curve calculates the number of cells needed to inoculate the co-culture. Co-cultivation started by inoculating 50 mL of MV media in a ratio of 20:1 with 20 × 10^6^ *B. subtilis* cells ml^−1^ and 1 × 10^6^ *S. cerevisiae* cells ml^−1^. Subsequently, the co-culture was incubated at 31.5°C and 180 rpm for 48 h. Samples of 1 mL were taken after 0, 4, 8, 24, and 48 h of cultivation and were centrifuged for 6 min at 6,000 *g*. Afterward, 800 μL of supernatant was discarded, and the remaining 200 μL of supernatant and cell sediment was diluted 1:2 for 8 h and 1:10 for 24 and 48 h samples. Finally, the samples were analyzed with flow cytometry to detect cell density and *B. subtilis* fluorescence (see the following subsection). Co-cultivation on solid media has been performed to show versatility and robustness. Co-cultivation started by fixing the ratio between *B. subtilis* 20 and *S. cerevisiae* cells in MV liquid media (Carl Roth, Karlsruhe, Germany) to 20:1 as described above and dropping 8 μL of made culture onto MV agar (Carl Roth, Karlsruhe, Germany) in a 6-well plate (transparent walls, clear bottom, Greiner Bio-One, Frickenhausen, Germany). The plate was dried under sterile conditions for 3 min to prevent a cell smear and afterward incubated for 24 h at 31.5°C. Fluorescence stereo microscopy of the grown colonies was performed at the start of incubation and after 24 h. The used microscope consisted of a Leica M205 (Leica, Wetzlar, Germany) FA stereo microscope equipped with a standard GFP filter (Leica, Wetzlar, Germany) (excitation 480 nm; emission 510 nm) and a Leica DFC3000G camera (Leica, Wetzlar, Germany). The acquired microscope images were analyzed using the Fiji ImageJ open-source platform (open-source platform developed by National Institutes of Health, Bethesda, MD, United States) (Schindelin et al., 2012) to eliminate background fluorescence and create merged images.

### 2.7 Flow cytometry

Flow cytometry was performed with samples taken from co-cultures of *B. subtilis* and *S. cerevisiae* by using CyFlow SL (Sysmex Partec GmbH, Görlitz, Saxony, Germany) equipped with a solid-state laser (488 nm). The emission filter IBP 527 nm was used. Co-culture samples were mixed well, and 10 μL of the sample was added to 2 mL of sheath fluid (Sysmex Partec GmbH, Görlitz, Saxony, Germany). The suspension was vortexed and forward scatter (FSc), side scatter (SSc), and fluorescence of the cells were measured in each sample (Supplementary Table S4). The FloMax software (version 2.52) (Sysmex Partec GmbH, Görlitz, Germany) was used to perform plotting of the FSc against SSc to visualize cell populations. Cell density for *B. subtilis* and *S. cerevisiae* was estimated by counting the cells (detected particles) within the populations and calculation by the equation in the appendices (Supplementary Equation S1). To analyze the fluorescence signal of *B. subtilis*, a population with a lower FSc value was plotted against the measured fluorescence signal (arbitrary unit [AU]). The mean *B. subtilis* population fluorescence value was calculated and corrected by the mean media blank value (see Supplementary Equation S2).

## 3 Results

### 3.1 Production of CSF and PhF in yeast

**Figure 2:**
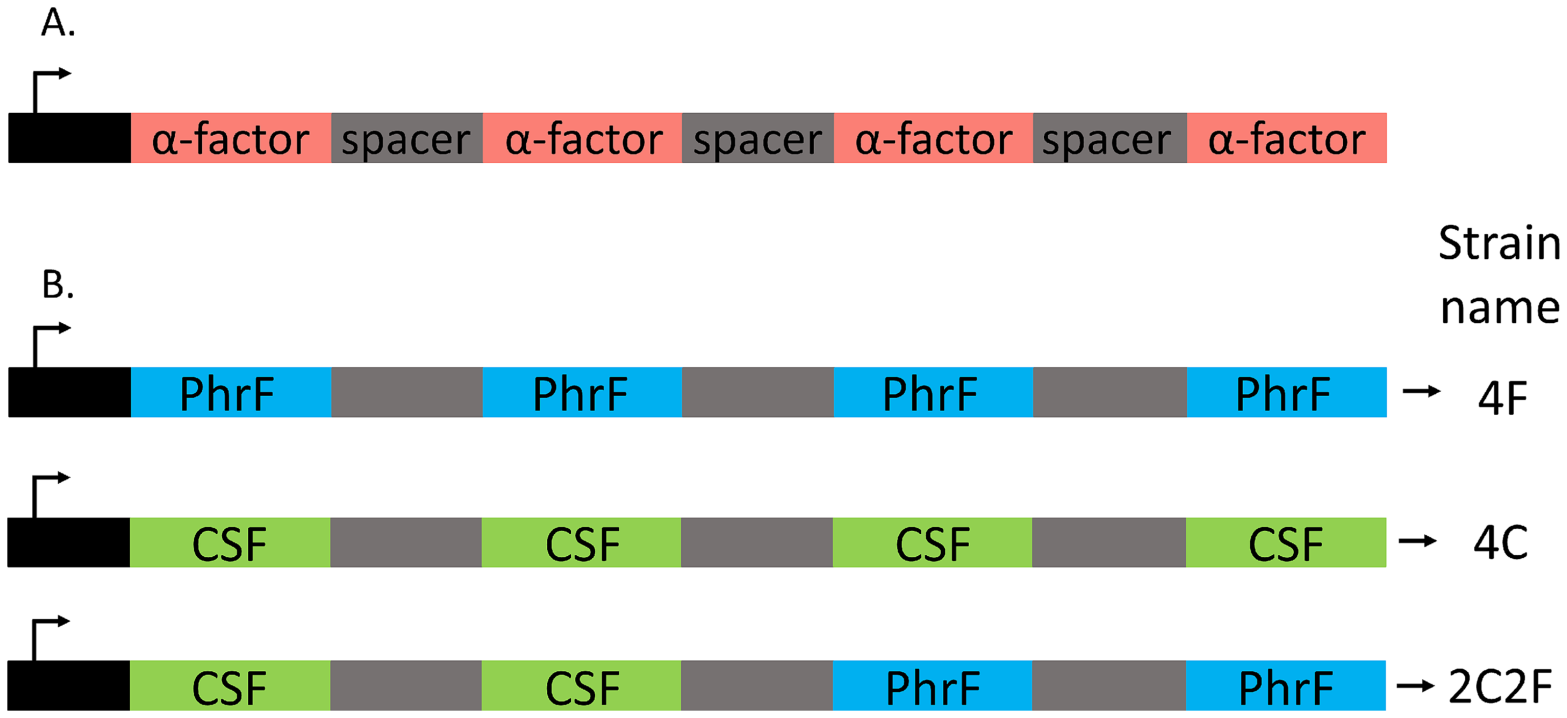
Artificially synthesized constructs for the heterologous production of competence and sporulation factor (CSF) and PhrF in *S. cerevisiae*. **(A)** Structure of the native *MFα1* gene in *S. cerevisiae* coding for four *α-*factor (red) pheromones. **(B)** Engineered cassettes are integrated into the vector p425GPD and transformed into *S. cerevisiae* for heterologous production of CSF (green) and PhrF (blue) peptides. Strain 4F is predisposed to produce four PhrF peptides; 4C, four CSF peptides; 2C2F, two CSF and two PhrF.

**Figure 3:**
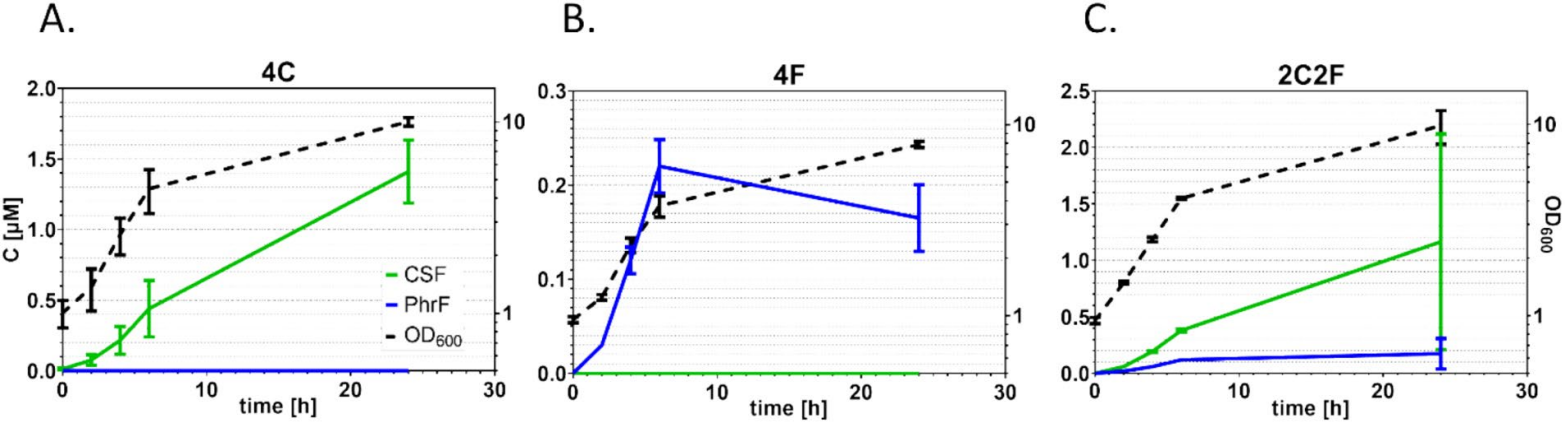
Peptide production in *S. cerevisiae* expression. *S. cerevisiae* culture growth (black dotted line) and supernatants were analyzed by mass spectrometry to determine the concentration of heterologous produced competence and sporulation factor (CSF) (green line) and PhrF (blue line). The left *y*-axis shows the peptide concentration, while the right *y*-axis shows the optical cell density measured at 600 nm. Growth and produced peptide concentrations of strain 4C **(A)**, 4F **(B)**, and 2C2F **(C)** are presented. Experiments were performed as duplicates after cultivation in MV media. Error bars represent the standard deviation from the mean value.

To develop an interkingdom communication system between yeast and bacteria, *S. cerevisiae* BY4741 ∆*bar1* strains that produce quorum-sensing peptides PhrF and CSF were constructed (Table 1). Since PhrF and CSF are expressed and transported outside the yeast cell, we used the expression principle for the natively produced small signal peptide *α*-factor (*MFα1* gene). Here, we replaced the coding sequence of *α*-factor, which is between signal and spacer sequences, with PhrF and CSF coding sequences (Figure 2). For the heterologous expression of bacterial peptides, we developed three *S. cerevisiae* strains that produce the desired peptides: 4C, 4F, and 2C2F, respectively (Figure 2). Strain 4C contains four CSF coding sequences in one construct; the 4F strain consists of four PhrF coding sequences, while strain 2C2F includes two CSF and two PhrF coding sequences. All constructs were cloned into and expressed via the vector p425-GPD. Next, we characterized the expression of the developed *S. cerevisiae* producer strains. Expression studies were performed in MV-media, and supernatants were analyzed with LC/MS–MS. In parallel, the optical density of cultures was measured to follow the growth of the strains. Both bacterial peptides can be produced successfully by the several yeast strains as indicated by the LC/MS–MS results, shown in Figure 3 and Supplementary Table S5. Furthermore, the concentration of the produced peptides rises with increasing optical density, which is expected due to the utilization of the constitutive *GPD* promoter that drives peptide expression. The concentration of produced CSF reaching 1.5 μM is significantly higher than that of PhrF, which reaches 0.2 μM. Interestingly, the concentration of CSF and PhrF is not significantly lower in the 2C2F strain compared to the 4C and 4F strains, indicating that the number of peptide coding sequences in the transcription cassette (see Figure 2) is not a limiting factor in peptide production.

### 3.2 Construction of *Bacillus subtilis* reporter strain

**Figure 4:**
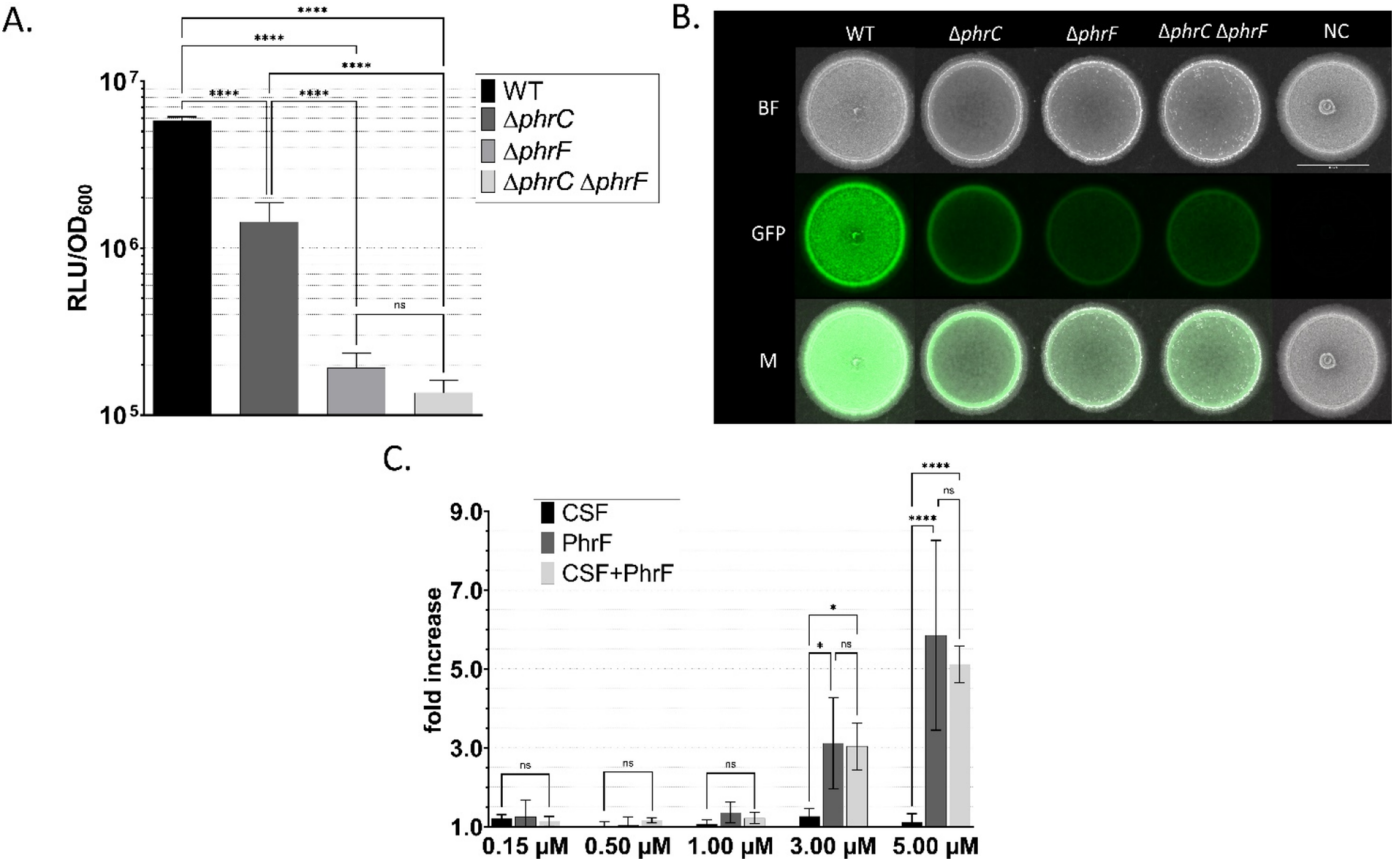
Characterization of *B. subtilis* P*_srfAA_* promoter activity in response to CSF and PhrF. *B. subtilis* P*_srfAA_*-*lux*ABCDE **(A,C)** and *B. subtilis* P*_srfAA_*-*sf*GFP **(B)** strains with *phrC-phrF* gene deletions were tested to monitor the promoter activity via the measurement of bioluminescence or fluorescence. A Maximum relative luminescence unit normalized with optical density (RLU/OD_600_) of the late exponential/early stationary phase of luciferase assay from *B. subtilis* P*_srfAA_*-*lux*ABCDE strains is presented. Statistical difference was determined using one-way analysis of variance (ANOVA) followed by Bonferroni’s multiple comparisons test for pairwise comparison between wild-type strain and mutants. **(B)** Fluorescence signals of *B. subtilis* P*_srfAA_*-*sf*GFP on solid media measured with a fluorescence stereo-microscope. Scale bar equals 5 mm; BF, bright field; GFP, green fluorescence channel; M, merged image; NC, negative control. **(C)** Fold changes in bioluminescence of strain *B. subtilis* P*_srfAA_*-*lux*ABCDE ∆*phrC* ∆*phrF* after the addition of synthetic CSF, PhrF, and mixed CSF/PhrF. Fold increase was calculated as the difference between the maximum relative luminescence units (RLU) of the induced and non-induced strain’s late exponential/early stationary phase. Statistical significance was determined using two-way ANOVA followed by Tukey’s multiple comparisons test to do a pairwise comparison between different inducing concentrations. All experiments were performed in MNGE liquid or on solid media (*n* ≥ 3). Error bars represent standard deviation from the mean value; ns, not significant; * *p* < 0.05; **** *p* < 0.0001.

After we successfully established *S. cerevisiae* quorum-sensing producer strains, developing *B. subtilis* PhrF/CSF-sensing strain was the next step to reach our goal of interkingdom signaling between eukaryotes and prokaryotes. First, a peptide-sensitive strain is determined by a lower reporter promoter activity than the wild type. This is necessary to create sensitivity and measurable responsiveness to the induction with heterologously produced CSF and PhrF because these are physiologically relevant peptides, which have their predominant function in fine-tuning quorum sensing-dependent gene expression and protein activity. Hence, no major changes in the wild type’s promoter activity are expected when induced with artificial CSF and PhrF peptides. Second, we evaluated, which modified strain shows the biggest increase in promoter activity after induction with CSF and PhrF. As already known, CSF and PhrF indirectly activate the transcriptional regulator ComA, which implies the use of gene promoters directly affected by the signaling pathway, for example, gene *srfAA*. Thus, the P*_srfAA_* promoter sequence was fused with *luxABCDE* bioluminescence or *sf*GFP fluorescence reporter genes to quantify promoter activity (Figure 4). We can confirm that introducing single and double deletions of genes responsible for the production of CSF and PhrF will decrease the activity of P*_srfAA_* (Bongiorni et al., 2005).

### 3.3 Measurement of reporter strain activity

We measured promoter activity in liquid media experiments with plate reader assays, while a fluorescence stereo microscope was used to measure promoter activity in solid media experiments (Figure 4). P*_srfAA_* activity is significantly lowered in a strain carrying a double deletion ∆*phrC* ∆*phrF* (> 50-fold compared to the wild type, Figure 4A), making the strain desirable for communication with *S. cerevisiae*. Furthermore, by stereo-microscopy images of colonies (Figure 4B) grown on MNGE solid media, we confirm that the strain carrying the double deletion ∆*phrC* ∆*phrF* has the lowest activity out of all tested strains (~4-fold). However, P*_srfAA_*, even including ∆*phrC* ∆*phrF* double deletions, is not entirely switched off due to the presence of other quorum-sensing peptides (e.g., PhrH) that influence the activity of the ComA transcriptional regulator (Smits et al., 2007; Wolf et al., 2016).

After confirming a successful decrease in P*_srfAA_* activity in a ∆*phrC* ∆*phrF* deletion mutant, we needed to establish an inducible reporter strain with artificially synthesized or heterologously produced peptides. A range of 150 nM up to 5 μM of synthetic CSF and/or PhrF induced the strain carrying the ∆*phrC* ∆*phrF* deletion (Figure 4C). A combination of the lowest promoter activity (deletions reduced the activity compared to the wild type) and highest sensitivity to the quorum-sensing peptides (highest induction with peptides) made strain ∆*phrC* ∆*phrF* desired for sensing the PhrF and CSF produced in yeast strains.

**Figure 5:**
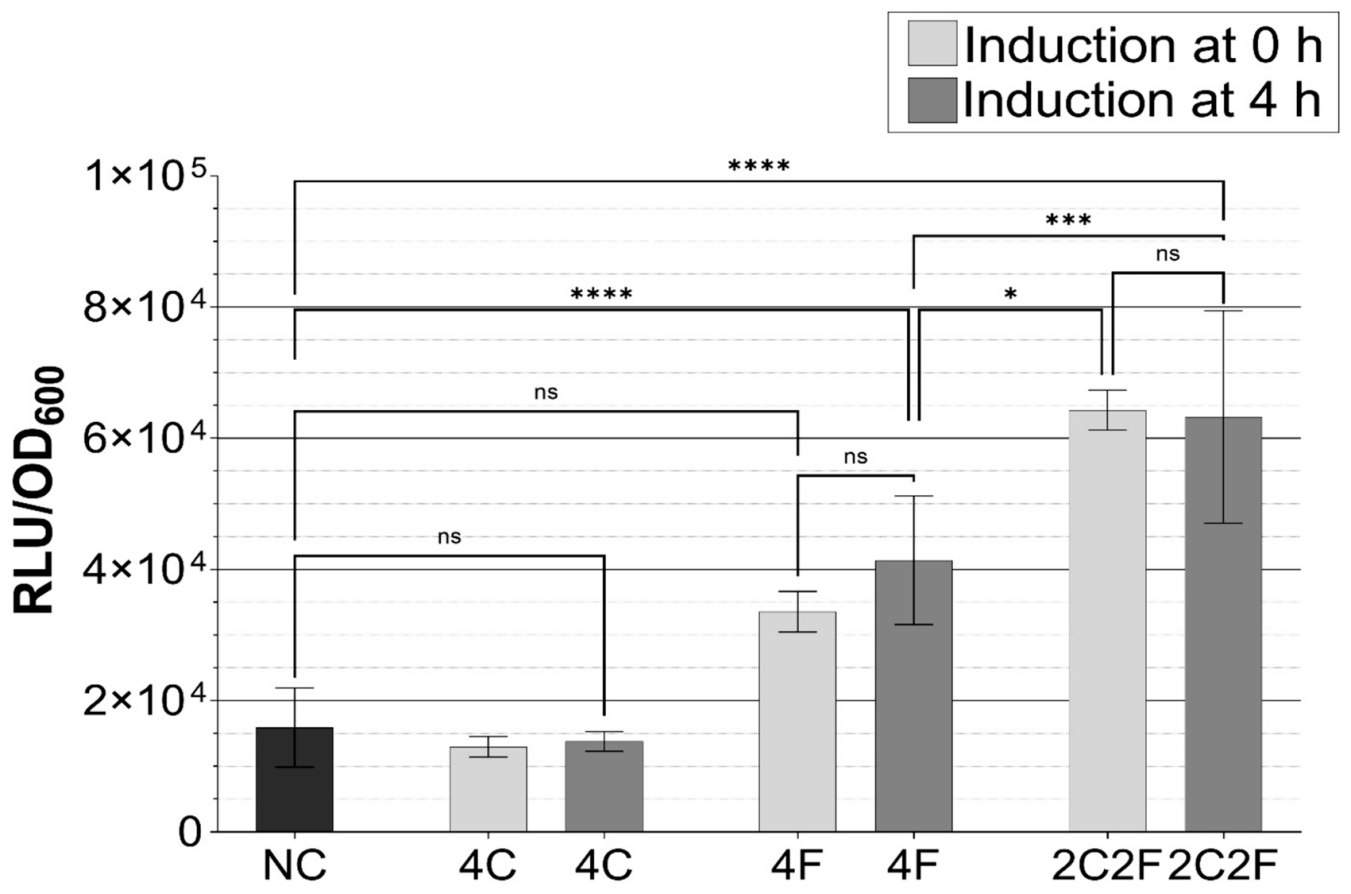
Promoter induction in *B. subtilis* P*_srfAA_*-*luxABCDE* ∆*phrC* ∆*phrF* with supernatants collected from *S. cerevisiae* expression strains. *B. subtilis* was induced with supernatants of *S. cerevisiae* producer strains (4C, 4F, and 2C2F) and negative control (*S. cerevisiae* empty vector) harvested after 24 h of cultivation. *B. subtilis* reporter strain was induced by adding 10 μL supernatant after 0 and 4 h of incubation. The experiments were performed in MV media as triplicates. Statistical difference was determined by using one-way analysis of variance (ANOVA) followed by Bonferroni’s multiple comparisons test to perform pairwise comparison between NC and peptide-producing strains. Error bars present standard deviation from the mean value; ns, not significant; * *p* < 0.05; *** *p* < 0.001, and **** *p* < 0.0001.

Finally, to test the ability of strain *B. subtilis* P*_srfAA_*-*luxABCDE* ∆*phrC* ∆*phrF* to sense the heterologously produced CSF and PhrF, we have induced luminescence in this reporter strain with supernatant of *S. cerevisiae* producer strains 4F and 2C2F harvested after 24 h of incubation (Figure 5). Furthermore, due to the *B. subtilis* natural competence pathway, which is activated in the late exponential and early stationary growth phase, we investigated how the induction time influences promoter activity as well. Results shown in Figure 5 nicely indicate that *S. cerevisiae* heterologously produced peptides maintained a biological activity and could activate the P*_srfAA_* promoter and thereby induce luminescence in *B. subtilis* P*_srfAA_*-*luxABCDE* ∆*phrC* ∆*phrF* (ranging from 2-fold to 5.5-fold). The higher fold increase obtained with CSF/PhrF from *S. cerevisiae* strain 2C2F compared to strain 4F indicates a possible synergistic effect of both produced peptides. Additionally, induction of reporter strain with heterologously produced peptides after 4 h gained a higher promoter activity than at the beginning of the measurements (Figure 5, 0 h). These results were expected since induction after 4 h occurs just before the start of the late exponential growth phase, which is time-wise closer to the state when ComA-activated promoters should reach maximal activity (Auchtung et al., 2006; Pottathil et al., 2008). These results suggest implications for potential successful co-cultivation experiments that took 48 h, as they show that *B. subtilis* P*_srfAA_* ∆*phrC* ∆*phrF* can be used as a reporter strain to detect CSF and PhrF produced by other strains at different time intervals.

### 3.4 Co-cultivation of *Saccharomyces cerevisiae* and *Bacillus subtilis*

**Figure 6:**
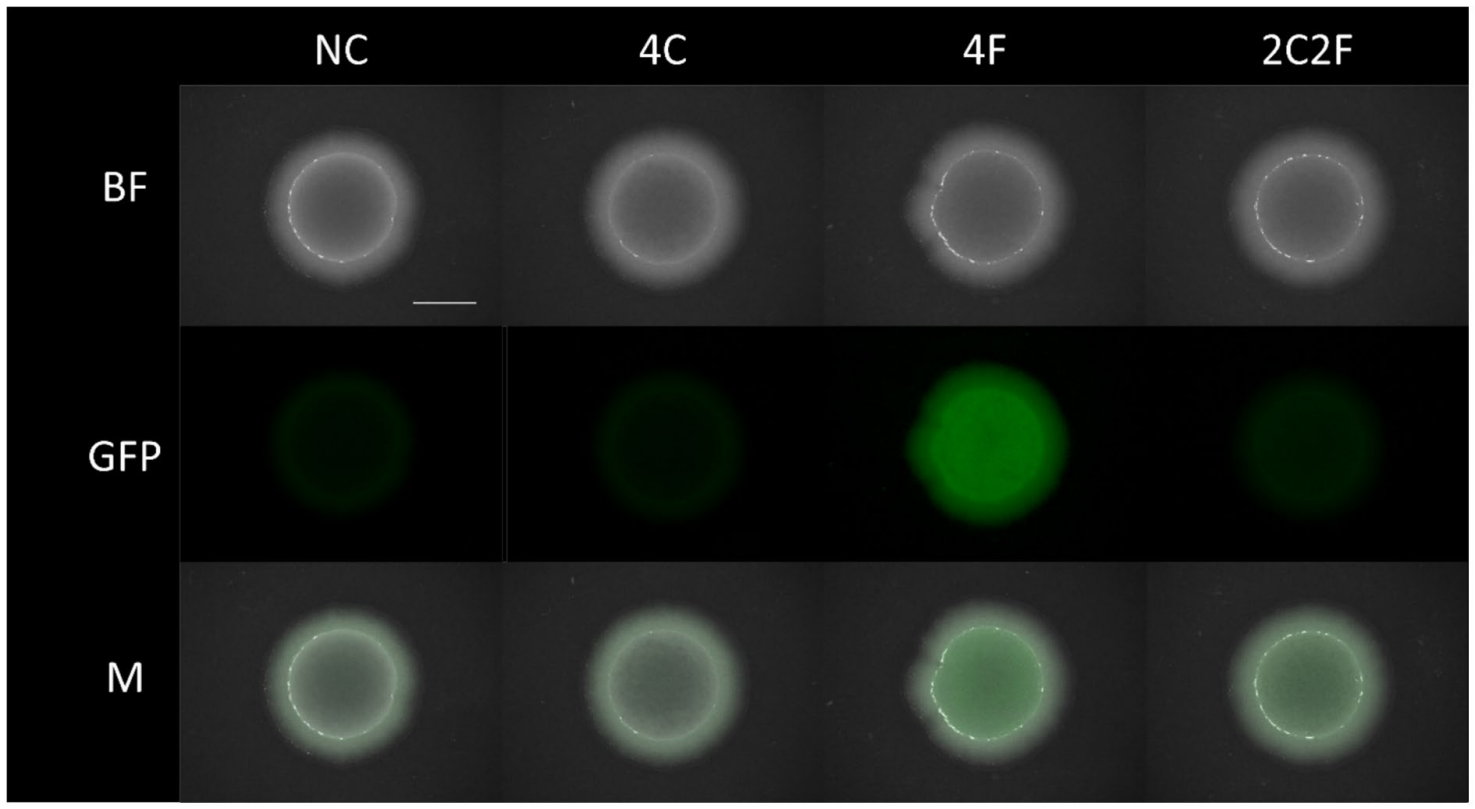
Co-cultivation of *B. subtilis* P*_srfAA_-sf*GFP ∆*phrC* ∆*phrF* with different *S. cerevisiae* peptide producing strains on solid media. Co-cultivation has been performed with *B. subtilis* P*_srfAA_-sf*GFP ∆*phrC* ∆*phrF* and *S. cerevisiae* strains NC, 4C, 4F, and 2C2F. Images were taken with a fluorescence stereo microscope after 24 h of co-cultivation at 31.5°C. Bright-field (BF) and fluorescence images were taken with Zoom 8×. The overlay of two channels is shown in M. All images were taken at the same exposure time (BF 4.80 ms, GFP 1.20 s) and intensity level (BF 30, GFP 49.9). The scale bar is equal to 5 mm. BF, bright field; GFP, green fluorescence channel; M, merged image; NC, negative control.

**Figure 7:**
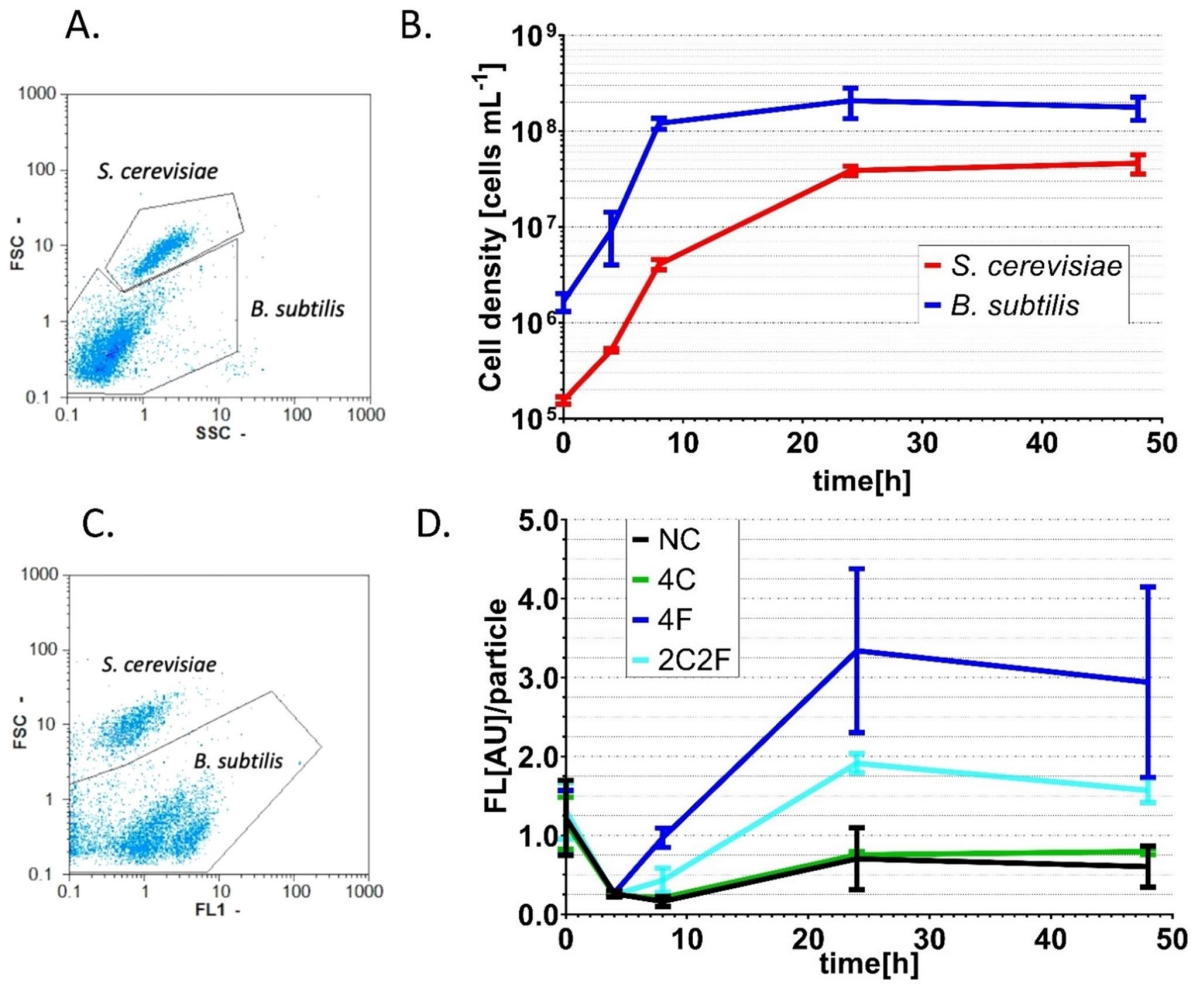
Co-cultivation of *B. subtilis* P*_srfAA_*-*sf*GFP ∆*phrC* ∆*phrF* and different *S. cerevisiae* peptide-producing strains. Co-culture samples taken at 0, 4, 8, 24, and 48 h were analyzed with a flow cytometer by measuring FSC and SSC. **(A)** Example of data gathered from the flow cytometer and “gated” signals from *B. subtilis* and *S. cerevisiae* cells. **(B)** Growth curves of *B. subtilis* reporter strain and *S. cerevisiae* producer strains that represent successful co-cultivation experiments. **(C)** Example of data gathered from a flow cytometer and “gated” fluorescence signal from *B. subtilis* cells. **(D)** The change of fluorescence per particle over the co-cultivation time. Presented fluorescence values are normalized to the blank values of the used media. Statistical significance was determined by using a two-way analysis of variance (ANOVA) followed by Tukey’s multiple comparisons test to compare NC and different peptide-producing strains over different time points. Statistically significant differences (*p* < 0.05) were observed after 24 and 48 h of co-cultivation between NC/4F and NC/2C2F. All experiments were performed in MV media as triplicates. Error bars represent the standard deviation from the mean value.

To prove the concept of interkingdom communication between yeast and bacteria, we performed co-cultivation experiments. Here, four different co-cultures were investigated to prove the biological response of *B. subtilis* to heterologously produced peptides from yeast. Due to a flow cytometer as an analytic device, the fluorescent *B. subtilis* P*_srfAA_*-*sf*GFP ∆*phrC* ∆*phrF* reporter strain was used, as the cells that can easily be distinguished from the yeast cells. Co-cultivation occurred with the reporter strain combined with *S. cerevisiae* strains NC (negative control, non-producer strain), 4F, 4C, and 2C2F in shaking flasks and on solid MV media, respectively. First, co-cultivation experiments were visualized on solid agar plates, and P*_srfAA_* promoter activity was determined by fluorescence measurement with a fluorescence stereo microscope (Figure 6). Negative control and *S. cerevisiae* producer strain 4C did not induce P*_srfAA_* promoter activity and, thereby, fluorescence in the *B. subtilis* reporter strain. The highest fluorescence signal in the *B. subtilis* reporter strain was reached in co-culture with *S. cerevisiae* producer strain 4F, which emphasizes the importance of PhrF. Following this analysis, we aimed to measure interkingdom signaling in the co-cultivation of *B. subtilis* and *S. cerevisiae* strains in liquid media. The first challenge in establishing the co-cultivation of *B. subtilis* and *S. cerevisiae* in shaking flasks was the determination of the growth parameters in which both strains will grow with similar growth rates to prevent outgrowing and biomass domination of one species over the other. For this reason, we have analyzed the growth behavior of the cells in co-cultivation. Samples were taken after 0, 4, 8, 24, and 48 h, cells were analyzed with a flow cytometer, and two populations were formed in the FSC-SSC diagram (Figure 7A). Population with lower FSC and SSC outline *B. subtilis* cells, while higher values correspond to *S. cerevisiae* cells. The growth curves of *B. subtilis* P*_srfAA_*-*sf*GFP ∆*phrC* ∆*phrF* and *S. cerevisiae* negative control strain in Figure 7B represent the growth behavior of cells in co-cultivations in this study. Cells are in the exponential growth phase for the first 8 h of co-cultivation and do not overgrow each other. After 8 h of cultivation, cells of both species enter the stationary growth phase, which is stable until the end of the experiment after 48 h (Figure 7B). After establishing robust co-cultivation conditions in liquid culture, we investigated the biological response of *B. subtilis* to heterologously produced peptides CSF and PhrF from yeast using the flow cytometer (Figures 7C,D). Additionally, to FSC and SSC, we used the FL1 channel to determine fluorescence to check promoter activity in the reporter strain induced by CSF and/or PhrF produced from yeast cells. Again, samples were analyzed over 48 h of co-cultivation, and an FSC-FL1 diagram was obtained showing two cell populations dependent on their size and fluorescence intensity (Figure 7C). From the start of the co-cultivation until 4 h later, the fluorescence signal is slightly decreasing. This is probably a result of overnight culture autoinduction, where other quorum-sensing peptides (e.g., ComX, PhrH) initiate ComA and thereby activate P*_srfAA_* (Rahmer et al., 2015; Smits et al., 2007; Treinen et al., 2023). After 4 h in co-culture with *S. cerevisiae* producer strains 4F or 2C2F, the fluorescence signal of the *B. subtilis* reporter strain increases and reaches its maximum after 24 h (Figure 7D). *S. cerevisiae* producer strain 4F induced the strongest fluorescence (4.7-fold) and P*_srfAA_* promoter activity in the *B. subtilis* reporter strain compared to the negative control. In correlation, *S. cerevisiae* producer strain 2C2F led to a 2.7-fold higher fluorescence in the reporter strain, indicating a more substantial influence of PhrF over CSF under co-cultivation conditions. On the contrary, yeast producer strain 4C did not induce any fluorescence in the *B. subtilis* reporter strain. The absence of fluorescence here was expected due to previous results shown in Figure 4B, where synthetic CSF peptide did not remarkably induce the reporter strain P*_srfAA_*-*luxABCDE* ∆*phrC* ∆*phrF*. The decrease in overall fluorescent values for the co-cultures of the *B. subtilis* reporter strain and yeast producer strains 4F and 2C2F, respectively, might be due to nutrient limitation. This observation aligns with the slight decrease in *B. subtilis* cell density at 48 h compared to 24 h (Figure 7B).

Taken together, *B. subtilis* induction with *S. cerevisiae* heterologously produced signal peptides CSF and PhrF, both in supernatants or direct co-cultivation, indicate the successful establishment of a robust controlled interkingdom communication.

## 4 Discussion

This work aimed to demonstrate the general feasibility of interkingdom communication between *B. subtilis* and *S. cerevisiae* via the quorum-sensing peptides CSF and PhrF. *S. cerevisiae* strain BY4741 ∆*bar1* was engineered to produce and secrete quorum-sensing peptides CSF and PhrF heterologously. In parallel, *B. subtilis* strain W168 was genetically modified to serve as a reporter, which produces measurable bioluminescent or fluorescent signals in response to CSF and PhrF peptides. Finally, the robustness of the interkingdom communication between yeast and bacteria was shown in co-cultivation studies.

Even though both quorum-sensing peptides CSF and PhrF were produced by *S. cerevisiae* using the same genetic construct, the concentration of produced peptides varied significantly. The lower concentration of PhrF might be caused by intracellular retention of this peptide due to an imbalanced transport between the endoplasmic reticulum, the Golgi apparatus, and cell membrane or due to a difference in peptide stability, indicating that CSF accumulation is the result of higher stability in MV culture media (Carl Roth, Karlsruhe, Germany) compared to PhrF peptide (Huang et al., 2018). Furthermore, similar CSF concentration in strain 4C and PhrF concentrations in strain 4F and strain 2C2F indicate that peptide production on transcriptional and translational levels is not a limiting factor, which further underlines the statement that concentration difference is due to the variance in peptide stability.

Our results showed, that synthetic CSF alone did not lead to significant P*_srfAA_* promoter activation in the Δ*phrC*Δ*phrF* mutant. Additionally, PhrF activated the promoter stronger than CSF, showing a higher sensitivity of the designed promoter toward PhrF than CSF peptide. These results are in line with already published data that indicate a special and concentration-dependent regulation role of CSF to ComA-dependent promoters (Solomon et al., 1996). CSF modulates the timing of competence and sporulation in a concentration-dependent manner. Additionally, the deletion of the *phrC* gene only slightly reduces P*_srfAA_* activity (Figure 4A) (Solomon et al., 1996). Furthermore, high concentrations (50–100 nM) of CSF lead to an inhibition of P*_srfAA_* promoter activity (Solomon et al., 1996). In the case of PhrF, our results confirm the previously published data that suggest an essential role of PhrF in terms of ComA-dependent gene expression stimulation. In the absence of *phrF*, the activity of P*_srfAA_* cannot be significantly stimulated by CSF (Auchtung et al., 2006).

The strain *S. cerevisiae* 2C2F showed a synergistic effect of both produced peptides CSF and PhrF due to the significantly higher induction compared to that of strain 4F. The higher impact and synergistic effect of mixed CSF and PhrF peptides on ComA-dependent promoters was first reported by Bongiorni et al. (2005). However, this synergistic CSF-PhrF effect could not be observed when the bacterial reporter strain was induced with the synthetic peptides or was grown under co-cultivation conditions. Co-cultivation in both liquid and solid media showed, contrary to the results shown in the luciferase assay, that peptides of the supernatants of yeast strain 4F activated the reporter strain *B. subtilis* P*_srfAA_-luxABCDE* ∆*phrC* ∆*phrF* approximately 1.5 times stronger than peptides from the yeast producer strain 2C2F. The absence of the CSF-PhrF synergistic effect might be due to the higher inducing concentration of CSF in co-cultivation studies. A 10-times diluted supernatant collected after 24 h from yeast producer strain cultures was used for the bioluminescence assay. Under co-cultivation conditions, yeast and bacteria are in direct contact for 48 h, and peptides can act directly. The result, that induction of the bacterial reporter strain 4 h after the start was higher than directly at the beginning of the assay (0 h) was expected by virtue of CSF and PhrF, as quorum-sensing peptides, being active in *B. subtilis* during the late exponential and early stationary growth phase (Comella and Grossman, 2005; Hahn and Dubnau, 1991; Yuan et al., 2021).

The induction of the *B. subtilis* reporter in co-cultivation with the yeast peptide producer strains on solid agar and in liquid media showed successful interkingdom communication between yeast and bacteria. The reason lay in the effective utilization of the peptides PhrF and CSF by the intrinsic *B. subtilis* quorum sensing pathway. Quorum sensing systems provide a flexible and dynamic mechanism for the regulation of gene expression in bacteria, especially when bacteria live in challenging environments (e.g., co-cultivation with other species) and need to respond to specific, not high-energy consuming changes in the environment (Liu et al., 2021). So far, interkingdom communication has been deeply studied in host-pathogen interaction, and applied systems were mostly conserved around the development of probiotics (Khan et al., 2023). Recently, the first programmed cross-kingdom communication was developed where separated bacteria *E. coli* cells transferred “messages” to *S. cerevisiae* through a nanodevice that served as “nanotranslator” (de Luis et al., 2022). The interkingdom communication in our approach placed two species directly in co-cultivation, significantly reducing the complexity and simultaneously brother the applicability of the programmed communication.

## Data Availability

The datasets presented in this study can be found in online repositories. The names of the repository/repositories and accession number(s) can be found in the article/Supplementary material.
