# Supplementary material for: Controlled interkingdom cell-cell communication between Saccharomyces cerevisiae and Bacillus subtilis using quorum-sensing peptides
Source: Front Microbiol. 2024 Dec 12;15:1477298. doi: 10.3389/fmicb.2024.1477298 (PMC11669912; doi:10.3389/fmicb.2024.1477298)
Supplement: Supplementary file 1 [file Supplementary_file_1.docx]

Supplementary Material

# Supplementary Tables

**Table S1**. MV media composition

| Components | 1 L of media |
| --- | --- |
| **macro elements (2x), pH 6** | 500 mL |
| **trace elements (375x)** | 2.67 mL |
| **vitamins (100x)** | 10 mL |
| **folic acid (20000x) (20 mg in 500 mL 0.5 mM NaOH)** | 100 µL |
| **glucose (10x) (20 % (w/v))** | 100 mL |
| **amino acids/**n**ucleic acid (20x)** | 50 mL |
| **demineralized H_2_O** | 337.3 mL |

| Components “Macro elements 2x” | weight/volume |
| --- | --- |
| **Na_2_ EDTA** | 0.04 g |
| **K_2_HPO_4_** | 3 g |
| **NaCl** | 0.5 g |
| **MgSO_4_ x 7 H_2_O** | 0.5 g |
| **CaCl_2_ x 2 H_2_O** | 0.1 g |
| **MES** | 27.3 g |
| **(NH_4_)_2_SO_4_** | 5 g |
| **Demineralized H_2_O** | ad 500 mL |

| Components “Trace elements 375x” | weight/volume |
| --- | --- |
| **ZnSO_4_ x 7 H_2_O** | 450 mg |
| **CoCl_2_ x 6 H_2_O** | 30 mg |
| **FeSO_4_ x 7 H_2_O** | 300 mg |
| **MnSO_4_ x 4 H_2_O** | 86.25 mg |
| **CuSO_4_ x 5 H_2_O** | 30 mg |
| **NaMoO_4_ x 2 H_2_O** | 41.25 mg |
| **H_3_BO_3_** | 101.25 mg |
| **KI** | 11.25 mg |
| **Demineralized H_2_O** | ad 100 mL |

| Components “Vitamins 100x” | weight/volume |
| --- | --- |
| **biotin** | 14 mg |
| **4-amino-benzoic acid** | 27.5 mg |
| **calcium pantothenate** | 135 mg |
| **nicotinic acid** | 135 mg |
| **inositol** | 3330 mg |
| **thiamine hydrochloride** | 135 mg |
| **pyridoxine hydrochloride** | 164 mg |
| **riboflavin** | 20 mg |
| **demineralized H_2_O** | ad 500 mL |

| Components “Amino- and Nucleic acids 20x” | weight/volume |
| --- | --- |
| **uracil** | 1.05 g |
| **l-histidine** | 1.35 g |
| **l-leucine** | 3.35 g |
| **l-lysine** | 3.20 g |
| **l-methionine** | 0.90 g |
| **l-glutamate** | 1.00 g |
| **l-phenylalanine** | 0.50 g |
| **l-serine** | 3.75 g |
| **l-threonine** | 2.00 g |
| **l-tryptophane** | 1.00 g |
| **l-tyrosine (+ 4.4 g NaOH to dissolve)** | 2.00 g |
| **demineralized H_2_O** | ad 500 mL |

**Table S2**. Plasmid containing *E. coli* strains used in this study

| Plasmid | Description | Source/Reference |
| --- | --- | --- |
| **p425GPD** | *E. coli* TOP10 F’ p425GPD | This study |
| **p425GPD-4F** | *E. coli* TOP10 F’ p425GPD-4F | This study |
| **p425GPD-4C** | *E. coli* TOP10 F’ p425GPD-4C | This study |
| **p425GPD-2C2F** | *E. coli* TOP10 F’ p425GPD-2C2F | This study |
| **174** | *E. coli* DH10β pBS1C | Radeck et al., 2013 |
| **179** | *E. coli* DH10β pBS3C*lux* | Radeck et al., 2013 |
| **TME4303** | *E. coli* DH10β pBS1C-P*_srfAA_*-*sf*GFP | This study |
| **TME4003** | *E. coli* DH10β pBS3C*lux* - P*_srfAA_* | This study |

**Table S3**. Primers used in this study

| Primer | Description | Primer sequence 5'−3' |
| --- | --- | --- |
| JK002 | Fwd primer | GACGGTAGGTATTGATTG |
| JK003 | Fwd primer | CGACGGATTCTAGAACTAG |
| JK004 | Rev primer | CCTCAATCTGTTGGACTTCAG |
| TM7022 | sfGFP fwd | GCCGCTTCTAGAGCGATAGGAGGTCTCGAATGGTTTCTAAAGGTGAAGCAGTGA |
| TM7023 | sfGFP rev | GTGGTCTGCAGGCTTTGTACAG TTCATCCATACCACCG |
| TM0749 | Check primer rev | AAAGGTCATTGTTGACGCGG |
| TM2262 | pAH328 check fwd | GAGCGTAGCGAAAAATCC |
| TM2263 | pAH328 check rev | GAAATGATGCTCCAGTAACC |
| TM2507 | pAH328 sacA back check fwd | GTCGCTACCATTACCAGTTG |
| TM2508 | pAH328 sacA back check rev | TCCAAACATTCCGGTGTTATC |
| TM6653 | oTV_TUD_01 | GATCGGAATTCGCGGCCGCTTCTAGAAGATTGAACGCAGCAGTTTGG |
| TM6654 | oTV_TUD_02 | GATCCTGCAGCGGCCGCTACTAGTAGCAGTGCTTCCAATCAAAAAACAG |
| TM6785 | Ppel_rew | agatcCTGCAGCGGCCGCTACTAGTaAATATAATAGCAAATTGAGGGACATTCAG |
| TM6786 | Ppel_forw_Drcluster | gatcGGAATTCGCGGCCGCTTCTAGAgGAAAAAACCAAAAGGCCAA |
| TM6797 | *phrC* fwd | CCAGCAGGAACTGAAACACAT |
| TM6798 | *phrC* rev | AGCATGGTTTGACGTATGAGC |
| TM6801 | *phrF* fwd | AGAGCTGGATCAAATGGAAGAAG |
| TM6802 | *phrF* rev | GATCTTCGTGTTGATTATGAGGG |
| TM6803 | *phrH* fwd | TTCAGCAGATGGAAGAAGATCAG |
| TM6804 | *phrH* rev | ATGTATGCCGTGATCTGGGTAG |

**Table S4**. Phase duration and measurement parameters applied on Flow cytometry

| Phase | Duration |  |  |
| --- | --- | --- | --- |
| **Pre-run** | 5 s |  |  |
| **Stabilize** | 60 s |  |  |
| **Run** | 80 s |  |  |
| Measuring parameter | Gain | Minimum limit | Maximum limit |
| **Forward scatter** | 160 | 40 | 999.9 |
| **Side scatter** | 122 | 10 | 999.9 |
| **Fluorescence**  **Filter: IBP 527** | 500 | 40 | 999.9 |

**Table S5**. MRMs for LC-MS/MS analyses of peptides

| Compound MRM | ESI mode | m/z Q1 | m/z Q3 | DP in V | EP in V | CE in V | CXP in V |
| --- | --- | --- | --- | --- | --- | --- | --- |
| **Analytes** | | | | | | | |
| CSF 1 | positive | 593.135 | 492.200 | 36 | 10 | 43 | 24 |
| CSF 2 | positive | 593.135 | 269.000 | 36 | 10 | 49 | 30 |
| PhrF 1 | positive | 604.202 | 491.200 | 71 | 10 | 43 | 22 |
| PhrF 2 | positive | 604.202 | 308.100 | 71 | 10 | 49 | 34 |
| PhrF 3 | positive | 604.202 | 268.000 | 71 | 10 | 47 | 30 |
| **ISTDs** | | | | | | | |
| CSF 1 | positive | 603,133 | 502,100 | 111 | 10 | 45 | 22 |
| CSF 2 | positive | 603,133 | 317,000 | 111 | 10 | 49 | 14 |
| PhrF 1 | positive | 614,202 | 501,200 | 10 | 10 | 43 | 24 |
| PhrF 2 | positive | 614,202 | 317,000 | 10 | 10 | 49 | 34 |
| PhrF 3 | positive | 614,202 | 596,200 | 10 | 10 | 33 | 28 |

# Supplementary Equations for Flow Cytometry

**Equation S1**. Determination of the cell density

$$Cell density\left[ cells {mL}^{-1} \right]=cell count*\frac{1}{Dilution}*\frac{1}{Sample volume}$$

**Equation S2**. Determination of fluorescence [AU]

$$FL\left[ AU \right]=\frac{\left( {cell count}_{sample}*{FL mean}_{sample} \right)-({cell count}_{blank}*{FL mean}_{blank})}{{cell count}_{sample}-{cell count}_{blank}}$$
